# Application of the Lalonde (Horizontal-Only Scar) Technique Reduction Mammoplasty in Oncoplastic Breast-Conserving Surgery for Asian Patients: A Case Report

**DOI:** 10.1055/a-2705-4640

**Published:** 2026-01-17

**Authors:** Ryohei Katsuragi, Hisamitsu Zaha, Ayako Koki, Norie Abe

**Affiliations:** 1 Department of Breast Surgery, Nakagami Hospital, Okinawa, Japan; 2 Department of Plastic, Reconstructive and Aesthetic Surgery, Faculty of Medicine, University of Toyama, Toyama, Japan

**Keywords:** oncoplastic breast-conserving surgery, Lalonde technique, oncoplastic, breast reduction

## Abstract

Oncoplastic breast-conserving surgery combines oncological resection with plastic surgery techniques, including reduction mammoplasty, to achieve effective cancer control and superior cosmetic outcomes.

Traditional approaches, such as vertical scar mammoplasty and inverted-T mammoplasty, have enabled extensive tumor resection but are often associated with the drawback of vertical scars. The Lalonde technique has been reported in Western patients to allow extensive partial mastectomy without leaving vertical scars. Here, we present the case of a 60-year-old Asian woman with macromastia and breast cancer who underwent reduction mammoplasty using the Lalonde technique. The postoperative outcomes revealed complete tumor resection alongside excellent cosmetic results. This case highlights the potential and possibilities of applying the Lalonde technique to Asian patients with ptotic breasts, and offers a promising and aesthetically superior option as one of the new oncoplastic surgery techniques.

## Introduction

Oncoplastic breast-conserving surgery (OBCS) is a surgical approach designed to enhance both oncologic and cosmetic outcomes, particularly in cases where conventional breast-conserving surgery results in suboptimal aesthetics. 1 By incorporating plastic surgical techniques, OBCS enables the resection of larger tumors while preserving the breast contour. This advancement has made breast-conserving surgery feasible for patients who would otherwise require mastectomy.

**Fig. 1:**
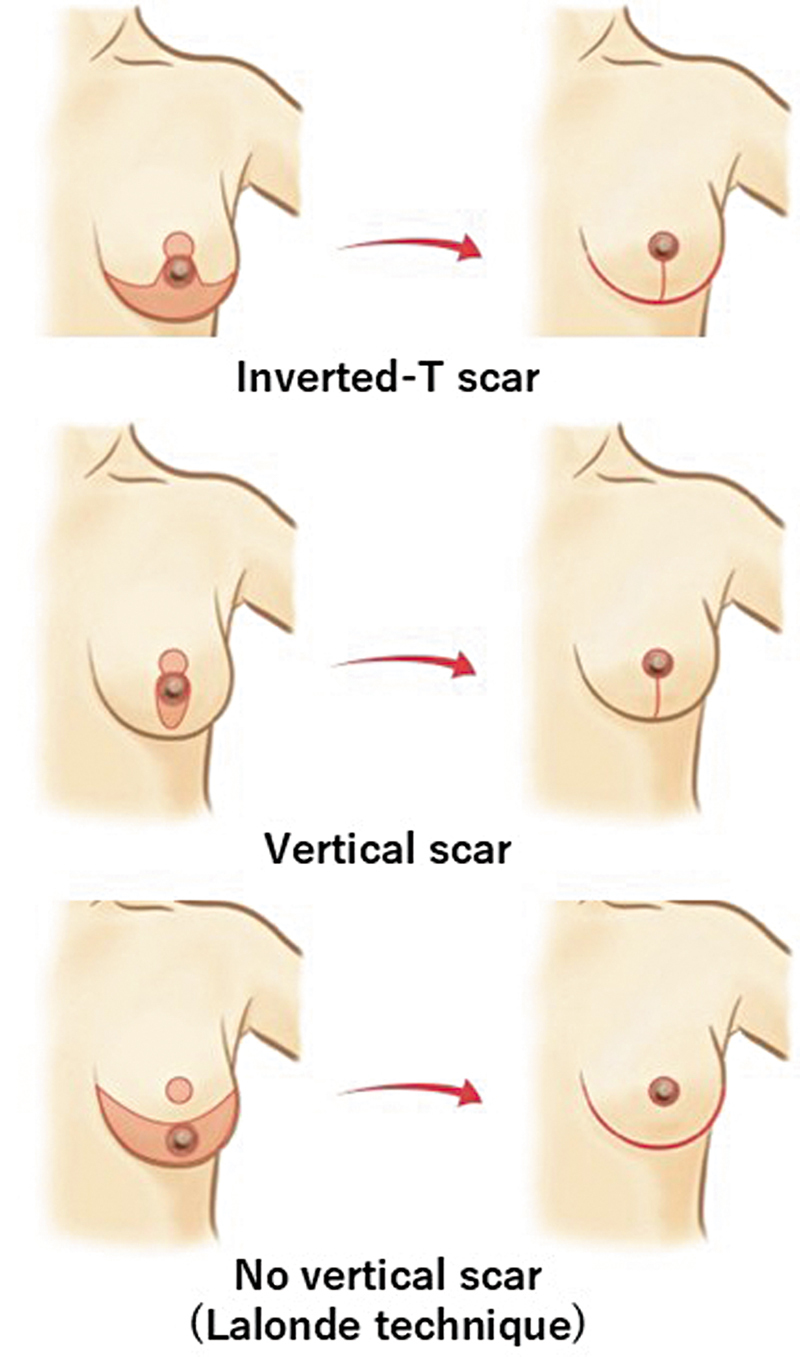
Surgical technique and scar pattern of reduction mammoplasty. Vertical scar mammoplasty and inverted-T mammoplasty leave visible vertical scars, while the Lalonde technique features horizontal scars along the inframammary fold (IMF). However, these scars are concealed by the breast when viewed from the front, giving the appearance of scars only around the areolar margin. This characteristic distinguishes the Lalonde technique.

OBCS techniques are broadly classified into volume displacement, which uses local breast tissue to fill a defect, and volume replacement, which involves tissue transfer from outside the breast. 2 Oncoplastic breast reduction (OBR), which is a volume displacement technique, has been widely adopted and commonly utilizes vertical scar mammoplasty and inverted-T mammoplasty. 3 Although these techniques enable extensive tumor resection, they leave vertical scars on the breast, which may compromise the final aesthetic outcome ( Fig. 1 ).

Here, we report the use of the Lalonde technique, 4 which avoids vertical scarring, in an Asian patient with ptotic breasts and breast cancer. This case report highlights the oncological safety and cosmetic advantages of this approach.

## Case

**Fig. 2:**
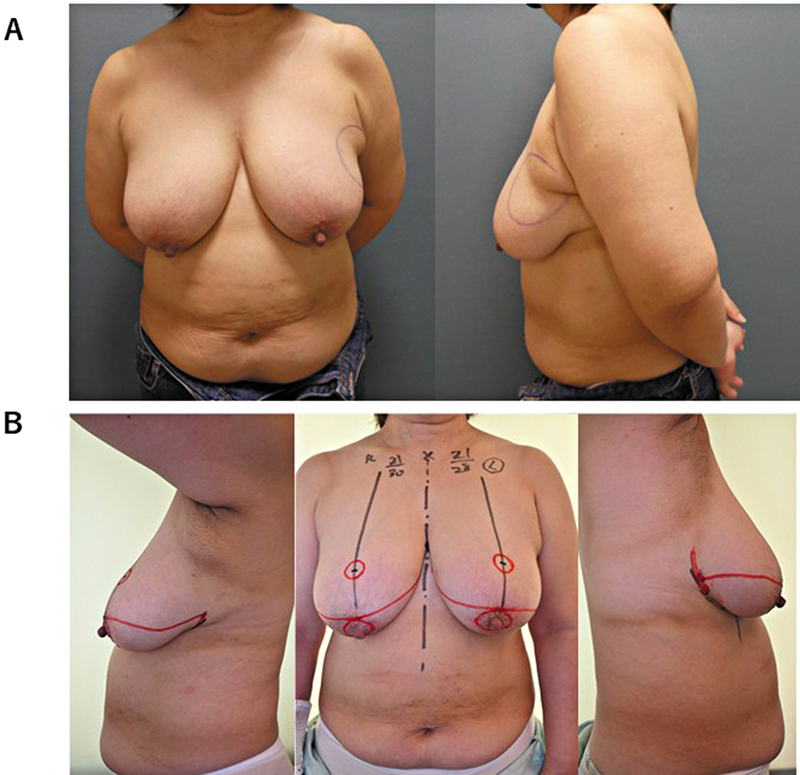
Preoperative photograph. ( **A** ) Breast cancer is localized in the upper outer quadrant of the left breast. The resection margin was marked to include the accessory axillary breast tissue. ( **B** ) The patient had macromastia with ptosis, with a sternal notch-to-nipple (SNN) distance of 30 cm on the right and 28 cm on the left. The upper incision was designed to extend from the medial IMF through the superior areolar margin to the lateral inframammary fold (IMF), while the lower incision followed the IMF from the medial to the lateral points. The original areola was excised at 40 mm, and the new areola was designed at a diameter of 25 mm and positioned at an SNN of 21 cm.

**Fig. 3:**
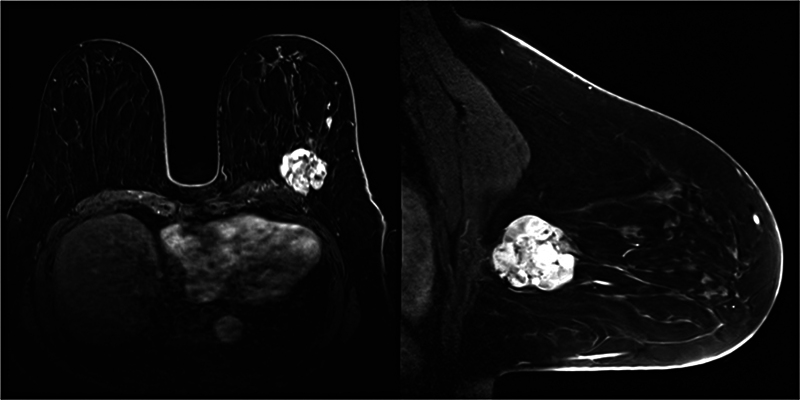
Preoperative contrast-enhanced breast MRI. A 43-mm lobulated tumor was identified in the upper outer quadrant of the left breast. The contrast-enhanced pattern displayed a fast-washout pattern with cystic structures observed within the tumor.

A 60-year-old woman (height, 151.2 cm; weight, 64.5 kg; body mass index [BMI]: 28.2 kg/m ^2^ ) presented with a palpable mass in her left breast ( Fig. 2A ). Core needle biopsy confirmed mucinous carcinoma (histological grade 2: estrogen receptor-positive, progesterone receptor-positive, human epidermal growth factor receptor 2 1 + , K _i_ -67 13%). The patient had grade III breast ptosis according to the Regnault classification, with sternal notch-to-nipple (SNN) distances of 30 cm on the right side and 28 cm on the left side ( Fig. 2B ). Imaging indicated a 43-mm cystic mass in the upper outer quadrant of the left breast with a fast washout pattern ( Fig. 3 ). Positron emission tomography/computed tomography indicated no distant metastasis, and the tumor was classified as cT2N0M0. The patient underwent left breast-conserving surgery with sentinel lymph node biopsy, axillary lymph node dissection (owing to the presence of positive nodes), and contralateral breast reduction for symmetry. Both procedures used the Lalonde technique.

### Surgical Technique

**Fig. 4:**
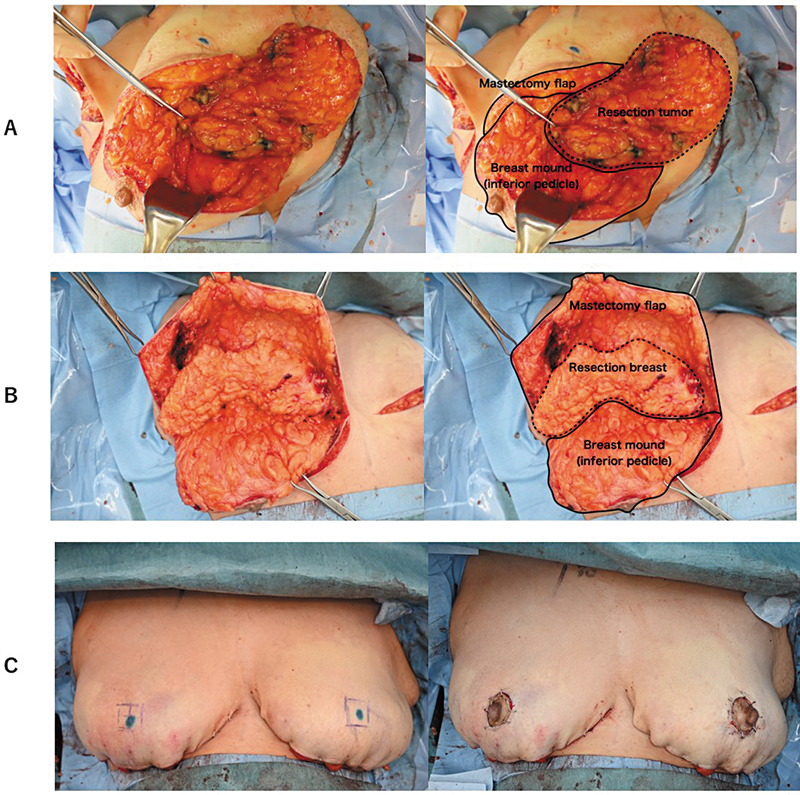
Intraoperative findings. ( **A** ) Left breast: A superior segment resection of 265 g was performed. The mastectomy flap was preserved with a thickness of at least 15 mm, and the nipple–areolar complex was vascularized using the inferior pedicle. ( **B** ) Right breast: A tumor weighing 300 g, including accessory breast tissue in the upper outer quadrant, was excised. Similar to the left breast, the mastectomy flap was preserved with sufficient thickness except directly over the tumor. The resection margins were roughly approximated with simple sutures. ( **C** ) After confirming breast shape in the sitting position, a 25-mm circular area was excised at the sternal notch-to-nipple (SNN) distance of 21 cm within the mastectomy flap, and the original areola (40 mm) was sutured into place.

Preoperative marking was performed with the patient in a standing position. The incision design included an upper incision from the medial inframammary fold (IMF) through the superior areolar margin to the lateral IMF and a lower incision along the IMF. The tumor localization was confirmed using ultrasonography, and the axillary accessory tissue was marked for resection. Under general anesthesia, a mastectomy flap was created, preserving at least 15 mm of the subcutaneous fat. The left breast was treated by removing a 300-g tumor-containing specimen ( Fig. 4A ), followed by sentinel lymph node biopsy and axillary dissection (levels I and II). Similarly, the contralateral breast underwent 265-g superior segment resection ( Fig. 4B ). The lower breast skin was de-epithelialized while preserving the areola (diameter, 40 mm). The new nipple position was set at an SNN of 21 cm by excising a 25-mm circular area in the mastectomy flap for areolar exposure ( Fig. 4C ). The surgical time was 2 hours and 55 minutes, with 165 mL blood loss.

**Fig. 5:**
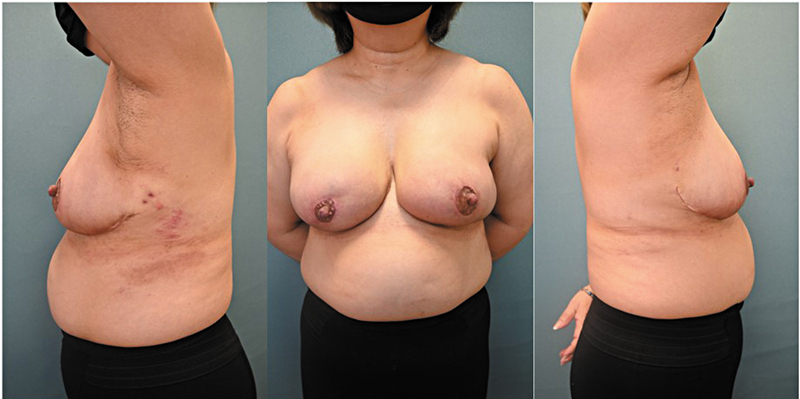
One-month postoperative photograph. No vertical scars are visible in the frontal view, and the incision appears confined to the areolar margin. The shape of both breasts is well-maintained.

The patient recovered uneventfully and was discharged on postoperative day 5. Pathological examination confirmed negative margins. At the 1-month follow-up, the patient showed no necrosis or atrophy, and the cosmetic outcome was excellent ( Fig. 5 ). Adjuvant endocrine therapy and radiotherapy were initiated.

## Discussion

OBR is a surgical approach that reduces the breast size through relatively extensive partial resection, creating a new breast with enhanced aesthetic quality. 3 Unlike other OBCS methods that aim to restore the breast to its original shape, OBR distinguishes itself by further enhancing the breast form during cancer resection, resulting in a more aesthetically pleasing outcome. In contrast to cosmetic reduction surgery, OBR does not allow surgeons to freely select the resection site or amount based on their preference, thereby limiting the choice of the nipple–areolar vascular pedicle. This results in more restrictions compared with conventional reduction surgery. The rate of postoperative complications is comparable to that of standard reduction mammoplasty, making it a well-established and safe technique. 5 No differences in local recurrence or overall survival between OBR and mastectomy have been reported, highlighting its oncological safety, which is similar to that of standard partial mastectomy. 6 Compared with mastectomy followed by breast reconstruction, OBR offers lower costs 7 and substantially higher levels of breast satisfaction, psychosocial well-being, and sexual well-being. 8 Traditional methods, such as vertical scar mammoplasty and inverted-T mammoplasty, impart noticeable vertical scars when viewed from the front, detracting from an aesthetic outcome. In general, this is a more important issue for Asians, whose scars tend to be more noticeable than those in Caucasians.

An important advantage is that the Lalonde technique leaves a scar on the IMF; however, from the front, the scar is concealed by the breast, giving the appearance of only a periareolar scar. 4 When conventional methods are used, the areola is often pulled by a vertical scar, resulting in a shape that may not always be circular. The Lalonde technique applies uniform tension in a circular manner, thereby minimizing postoperative areolar deformation. Although this is the first reported case of this technique in an Asian patient with breast cancer, the lack of previous reports may be attributed to the relatively lower prevalence of large ptotic breasts among Asians compared with Caucasians, as well as the limited awareness of this technique among surgeons in Asian countries. Eligible Asian patients could benefit greatly from the application of this method.

This surgical technique is not suitable for all cases of breast ptosis, and breast and tumor factors should be considered. The procedure is suitable for macromastia with Grade II or III ptosis, where the SNN is 30 cm or longer; a minimum length of 5 cm is required between the bottom of the new nipple–areola complex and the pigmented native areola. 9 In the current case, the left SNN of 28 cm still allowed for the formation of a well-shaped breast mound. The distance from the upper edge of the areola to the new nipple depends on where the new nipple is set within the mastectomy flap. Even in cases where securing 50 mm is not feasible, modifications in the incision design can enable successful implementation of the technique. 4 Based on our experience and the findings of Lalonde et al, 4 it may be possible to expand the indications for this technique to include breasts that do not meet the criteria described by Venardi et al. 9

This technique can be considered for tumors located in the upper quadrant. In reduction mammoplasty, various vascular pedicle methods, such as the superior, 10 inferior, 11 or central mound pedicle methods, 12 are used to ensure blood supply to the nipple–areolar complex. The Lalonde technique relies on the inferior pedicle for vascularization, which limits its application when resecting tumors in the lower pole because this may compromise blood flow to the nipple–areolar complex. Venardi et al 9 identified tumors located in the “5 to 7 o'clock” region of the breast as absolute contraindications to this approach.

The aesthetic drawbacks of the Lalonde technique include a boxy breast contour and diminished projection in the reconstructed breast. These limitations can be addressed by incorporating additional liposuction of the lateral breast tissue and by using suturing techniques that avoid extending the incision laterally instead of gathering the tissue along the IMF to enhance the contour. To improve projection, quilting sutures at the base of the glandular tissue may be effective. 4 Limitations of this case include the short postoperative follow-up period. However, 1 month postoperatively, no necrosis or atrophy was observed. There was no traction of the areola toward the caudal side, as is often seen in vertical scars, and the scars were not noticeable in the frontal view. The patient was highly satisfied with the breast cancer resection and reduction mammoplasty, both of which resulted in minimal visible scarring. Follow-up will continue as part of the patient's oncological care, with revisions planned as necessary in response to concerns or symptoms. The significance of this case report lies in demonstrating that non-vertical scar OBR can be safely performed in breasts of the size presented in this case, which were not excessively large. Thus, the range of indications for this technique can be expanded; more cases and long-term follow-up studies are essential to establish the specific criteria for its application.

### Conclusion

OBR using the Lalonde technique can successfully overcome the aesthetic drawbacks of conventional methods by avoiding vertical scars and confining scars to the inframammary fold and areolar margin. This approach is particularly beneficial for Asians, who tend to have more noticeable scars. This first application in an Asian patient demonstrates its feasibility and aesthetic advantages, even for breasts smaller than those reported in Western patients, while maintaining oncologic safety. Further studies and long-term follow-up are required to define the precise range of indications for this method.
